# Management of patella maltracking after total knee arthroplasty: a systematic review

**DOI:** 10.1007/s12306-022-00764-9

**Published:** 2022-10-05

**Authors:** R. Ferri, V. Digennaro, A. Panciera, B. D. Bulzacki Bogucki, D. Cecchin, M. Manzetti, M. Brunello, C. Faldini

**Affiliations:** 1st Orthopaedic and Traumatologic Clinic, IRCCS Istituto Ortopedico Rizzoli, Via G.B. Pupilli 1, 40136 Bologna, Italy

**Keywords:** Patella maltracking, Total knee arthroplasty, Knee pain, Outcome, Review

## Abstract

**Purpose:**

Patella maltracking is among the most frequent causes of poor outcomes and early failure after total knee arthroplasty (TKA), with an incidence that ranges from 1 to 20%. Even if there is agreement between authors regarding the preoperative and intraoperative management of patella maltracking in TKA, less clear are postoperative conducts. The purpose of this systematic review is to summarize and compare surgical techniques used to treat patella maltracking after TKA.

**Methods:**

A systematic review of the literature was performed with a primary search on Medline through PubMed. The PRISMA 2009 flowchart and checklist were used to edit the review. Screened studies had to provide clinical, functional and radiological results and complications of the proposed treatment to be included in the review.

**Results:**

A total of 21 articles were finally included. Three main types of surgical procedures and other minor techniques have been identified to manage patella maltracking after TKA. The choice of the proper technique to use in the specific case depends on several factors, first of all the malpositioning of the prosthetic components.

**Conclusion:**

Patella maltracking after TKA represents a frequent and challenging problem for orthopedic surgeons. Treatments described in the literature are often able to correct an abnormal patellar tracking; nevertheless, authors report variable percentages of residual knee pain and dissatisfaction in re-treated patients. Therefore, it would be desirable to prevent the maltracking condition at the time of primary arthroplasty, using proper surgical precautions.

## Introduction

Total knee arthroplasty (TKA) has become the standard treatment for various disabling disorders of the knee, especially end-stage osteoarthritis, and has proven long-term success. Surgical technique and prosthetic design have evolved to produce consistent and excellent results. Nevertheless, dissatisfaction rates among patients vary from 11 to 25% [1–6].

Patellofemoral joint disorders are the most frequent causes of painful knee and early failure after TKA [7, 8]: patella maltracking is one the most common conditions with an incidence that range from 1 to 20% and is generally a consequence of intraoperative technical errors [9, 10].

Patella maltracking is defined as a displacement of the patella center to a pathological position and features conditions like excessive patellar tilt, subluxation or complete dislocation [11].

Patella maltracking after TKA can lead to anterior knee pain (especially during activities such as stair climbing or chair rising) [12], increased component wear (with higher risk of component loosening), patellar fracture and instability [13].

In TKA, the key for obtaining an optimal patellar tracking should focus on achieving the correct patellar position, maintaining a stable tibio-femoral joint at the same time [11, 14].

Patella maltracking can generally be attributed to patient-related factors, implant design or surgical technique [15]. Demonstrated patient-related factors are preoperative valgus alignment [12], weakness of the quadriceps muscle (particularly the vastus medialis oblique) [1], patellofemoral dysplasia and/or previous patellar subluxation episodes [15], a lateral patellar shift > 3 mm in axial radiographs [16, 17]. The effect of implant design on patellofemoral stability is well recognized: femoral components featuring symmetrical and shallow trochlear groove have been shown to create abnormal patellar kinematics and increase the risk of patella maltracking [15, 18].

Errors in surgical procedures are the most frequent causes of patella maltracking: residual valgus limb malalignment, patella alta, excessive internal rotation of the femoral and/or tibial component, valgus alignment of the femoral component, asymmetrical patellar resection, lateral positioning or excessive thickness of the patellar button, incorrect soft-tissue balancing and missing or insufficient lateral release if needed have all been shown to have a negative effect on patella tracking [2, 11, 13, 15, 19, 20].

Over the years, many authors have proposed technical solutions to prevent patella maltracking in TKA, as reported by numerous in vitro and in vivo papers. Main measures concern the adequate positioning of the prosthetic components [2, 21–30], prefer prosthesis with anatomical “patella-friendly” femoral component design [31–34], a staged lateral retinaculum release carried out step by step [35–40] or a lateral patellar osteotomy as an alternative to decompress the lateral ligamentous structures [41, 42], access the knee joint through a lateral parapatellar or subvastus approach, particularly in the valgus knee [43–47].

Even if there is agreement between authors regarding the preoperative and intra-operative management of patella maltracking in TKA, less clear are the postoperative conducts.

The literature provides many case-reports and case-series studies regarding this topic, describing surgical procedures as lateral retinaculum releases, medial soft-tissue reconstructions, proximal and/or distal realignment techniques of the extensor apparatus, partial or complete revision of prosthetic components. Nevertheless, the literature lacks a systematic collection of the aforementioned works, mostly concerning complications and adequate indications of these techniques in specific cases. The purpose of this systematic review is to summarize and compare indications, complications, clinical, functional, and radiological results of surgical techniques used to treat the patella maltracking after TKA.

## Material and methods

A systematic review of the literature was performed with a primary search on Medline through PubMed used the following key-words: ((total knee replacement OR total knee arthroplasty) AND (patella maltracking OR patella instability OR patella malalignment OR patella dislocation OR patella displacement OR patella shift OR patella tilt OR patella subluxation OR patella luxation)).

The inclusion criteria were: studies providing clinical, functional and radiological results and complications concerning the treatment of patella maltracking after TKA, specifically in postoperative management; retrospective or prospective clinical studies including randomized controlled trials, nonrandomized trials, cohort studies, case–control, case-reports and case-series studies with a minimum follow-up of 1 year; papers in English without any restriction on publication date. The exclusion criteria were: review articles; in vitro or experimental biomechanical or cadaveric studies; papers not in English; studies concerning preoperative and intra-intraoperative precautions to avoid patella maltracking in total knee arthroplasty; studies concerning the management of patella maltracking in unicompartmental knee arthroplasty (both femoro-tibial and patello-femoral arthroplasty).

One author applied the previously determined criteria to select potentially relevant papers. Articles were initially identified based on title and abstract: full-text versions of relevant trials were then obtained and evaluated. References of the identified articles were checked not to miss any further relevant articles. The PRISMA 2009 flow chart and checklist were considered to edit the review.

The Level of Evidence (LOE) of the studies was assigned based on the 2011 Oxford Centre for Evidence-based Medicine Levels of Evidence.

The following data, when available, were extracted from the articles: Level of Evidence, number of patients, number of treated knees, mean age of patients, preoperative diagnosis (particularly if the postoperative treatment of patella maltracking regarded primary or revision TKA), main treatment, mean follow-up, the success rate in clinical, functional, and radiological tracking correction, complications occurred.

## Results

**Fig. 1:**
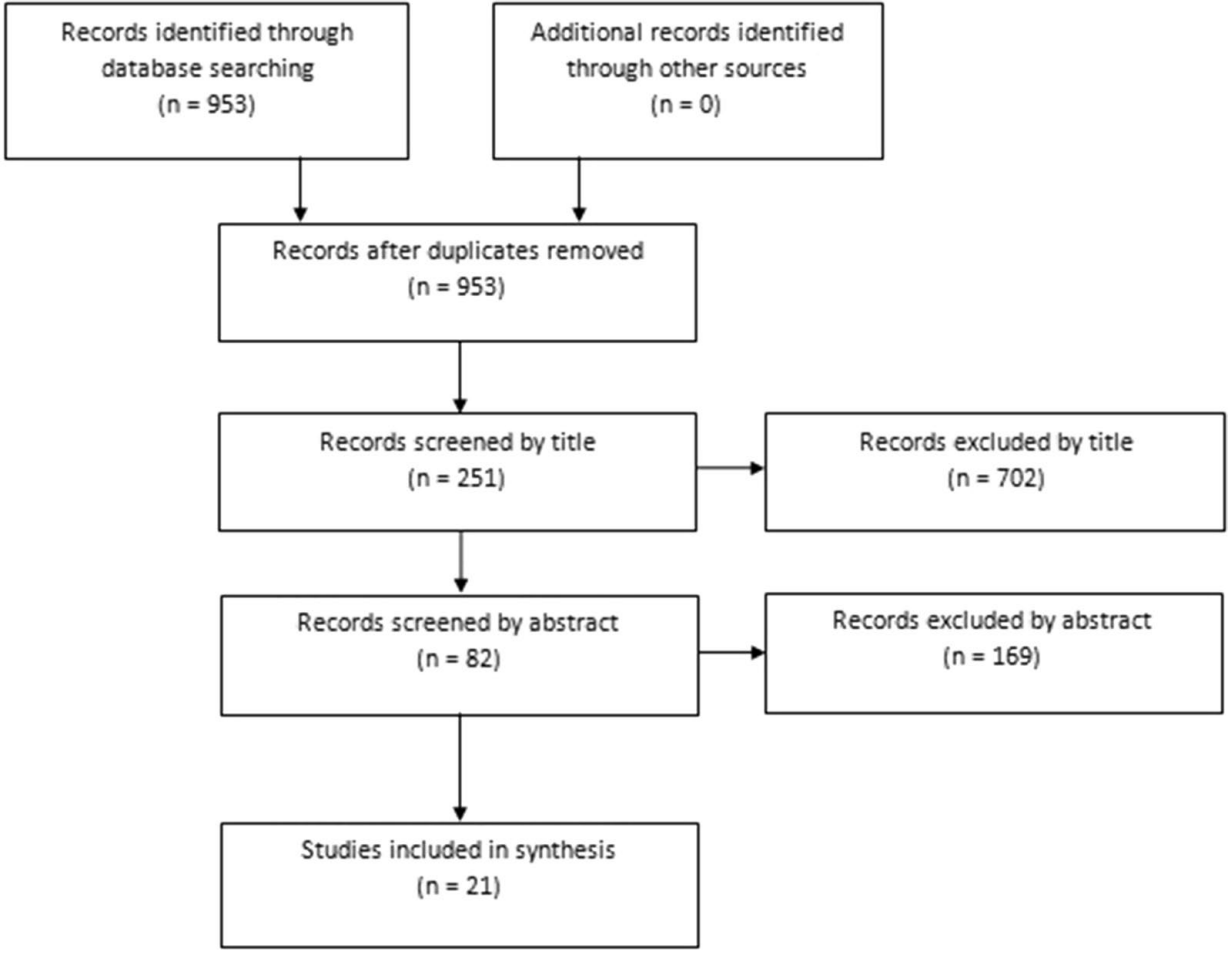
The PRISMA flow diagram illustrates the studies that have been identified, included, and excluded

A total of 21 articles were finally included in the systematic review. The PRISMA 2009 diagram illustrates the studies that have been identified, included, and excluded (Fig. 1). Table 1 describes data extracted from the included papers, and Table 2 summarizes the main surgical procedures used by authors to treat the patella maltracking after TKA.

**Table 1:** Summary of the data extracted from the included studies, presented in a chronological order based on the publication dates

Authors	LOE	N° knees	Mean age (years)	Preoperative diagnosis	Main treatment	Mean follow-up (months)	Radiological results	Clinical and functional results	Complications occurred
Merkow et al. 1985 [9]	IV	12	62 (46–70)	Patella maltracking after primary TKA	-10 proximal realignment (Insall procedure)-1 proximal realignment combined with components revision-1 lateral retinaculum release only	34 (24–57)	Patella was centrally located in the trochlear groove in 10 knees and slightly tilted laterally in 2	The results using The Hospital for Special Surgery knee-rating scale were excellent in 10 knees and good in 2. The average score was 89 (range 84–92), an increase of 27 (range 18–40) from the preoperative score. Pain was relieved in all but one patient, that had a mildly painful click during active extension	1 superficial skin necrosis healed with local care; 1 horizontal fatigue fracture of the patella
Grace et al. 1987 [48]	IV	25	72 (60–86)	Patella maltracking after primary TKA	-14 proximal realignment (Insall procedure)-9 combined proximal and distal realignment (modified Hauser procedure) -2 components revision	50 (24–125)	20 knees had a normal patellar tracking, 5 had recurrent subluxation	The average Hospital for Special Surgery knee-rating scale in patients treated with proximal realignment was 72 (range 39–88), with combined realignment 79 (range 55–93), with component revision 86 (range 84–88). Pain was reduced in all knees, 16 had no pain, 5 mild pain, 4 moderate pain	Flexion decreased from an average of 105° (range 80°–130°) to 93° (range 45°–130°); 2 patellar tendon ruptures; 1 loss of staple fixation; 2 late deep infection necessitated component removal and arthrodesis
**Bocell** et al. 1990 [49]	IV	2	/	Patella maltracking after primary TKA	Arthroscopic lateral retinaculum release	18–22	All knees achieved normal patellar tracking	All patients had clinical and functional improvement without a specific score system declared	None
**Johnson** et al. 1990 [50]	IV	5	68.5 (57–81)	Patella maltracking after primary TKA	Arthroscopic lateral retinaculum release and lysis of parapatellar adhesions	16 (3–32)	All knees achieved normal patellar tracking	The mean preoperative knee score* was 48, and improved to 83, representing a 73% improvement. The mean preoperative pain score was 25 (out of 50) and improved to 45, representing an 80% improvement	None
Kirk et al. 1992 [51]	IV	15	67 (54–77)	Patella maltracking after primary TKA	Lateral retinaculum release and medialization of the tibial tubercle (modification of the Trillat procedure)	24 (12–48)	Patella properly positioned in the femoral component groove in all but one patient, had some residual patellar tilt but there was no subluxation	The results using the Hospital for Special Surgery knee-rating scale averaged 82 (range 61–95). 11 patients rated good or excellent, 1 rated fair, 2 rated as failures (1 had persistent pain of unknown origin, 1 had a nonunion of the osteotomy)	1 nonunion of the osteotomy; 1 patient had loss of flexion (preoperative 90° preoperative to 80° postoperative); 1 late-onset infection
Chin et al. 2004 [52]	IV	39	68 (27–91)	Patella maltracking after primary TKA	-12 lateral retinaculum release only -14 lateral retinaculum release and advancement of the VMO -1 advancement of the VMO only -4 V–Y quadricepsplasty and lateral retinaculum release -7 V–Y quadricepsplasty, lateral retinaculum release and advancement of the VMO -1 varus tibial osteotomy (to correct a 30° valgus malunion of the proximal tibia)	38,4 (24–84)	All but one knees achieved normal patellar tracking, 1 had recurrent dislocation with an associated quadriceps tendon rupture	The mean prerevision Knee Society Score was 34 (range 0–74) and the mean prerevision Knee Society function score was 35 (range 0–100), the mean postrevision scores were, respectively, 77 and 54, with average improvements 43 and 19	1 recurrent dislocation with quadriceps tendon rupture; 1 lateral skin flap necrosis; 1 patella fracture and osteonecrosis; 1 superficial wound infection; 1 deep infection
Campbell et al. 1995 [18]	IV	14	70.4 (38–88)	Patella maltracking after primary TKA	- 6 lateral retinaculum release - 4 lateral retinaculum release and medial plication - 2 lateral retinaculum release and tibial tubercle realignment -1 tibial component revised -1 polyethylene tray revised and distal realignment	14–44	Undeclared	10 patients had a resolution of their symptoms (undeclared score system), 2 some improvement, 1 no change and 1 had a deep infection	1 deep infection managed by debridement and long-term antibiotics
Whiteside et al. 1997 [53]	IV	31	/	Patella maltracking after primary TKA	a. 10 modified Roux-Goldthwait procedure b. 3 medial transfer of the medial 1/2 of the patellar tendon c. 18 medial tibial tubercle transfer	12	All knees achieved normal patellar tracking. No late patellar subluxations or dislocations have occurred	All patients had clinical and functional improvement without a specific score system declared	a. none b. none c. 3 hematomas of which 2 required surgical evacuation and in 1 late-onset deep infection developed that required removal of the implants, debridement and revision arthroplasty
Asada et al. 2007 [54]	V	1	82	Patella maltracking after primary TKA	MPFL reconstruction	24	The knee achieved normal patellar tracking	The patient had clinical and functional improvement without a specific score system declared	none
Incavo et al. 2007 [55]	IV	22	69 (42–88)	Patella maltracking after primary TKA with one of more malrotated components	Full-components revision (2 femoral components only)	/	All but 2 patellae tracked centrally	The prerevision to postrevision Knee Society Score improved from 42 (range 10–62) to 77 (range 65–95), whereas the Knee Society function scores improved from 38 (range 5–60) to 49 (range 10–85). 3 patients had moderate pain, 2 had mild pain, the remaining had no pain	1 mediolateral laxity greater than 10°; 1 patella osteonecrosis because of patella remnant was 8 mm thick; 1 deep vein thrombosis
Price et al. 2009 [56]	IV	5	68.8 (60–76)	Patella maltracking after primary TKA	- 4 lateral retinaculum release, components revision and Fulkerson osteotomy -1 lateral retinaculum release and Fulkerson osteotomy only	29.7	All knees achieved normal patellar tracking	The average preoperative Knee Score was 70,5 that improved to 85.0. No patients complained of any pain	1 cellulitis secondary to venous stasis that resulted in a deep infection requiring irrigation and debridement with resolution
Nakajima et al. 2010 [57]	V	1	74	Patella maltracking after primary TKA	Elmslie-Trillat procedure (lateral retinaculum release, plication of the medial retinaculum, and medial displacement of the tibial tubercle)	12	Patella was slightly tilted laterally but there was no evidence of patellar maltracking or subluxation	The patient had clinical and functional improvement without a specific score system declared	none
Lakstein et al. 2010 [58]	IV	24	68 (53–83)	Patella maltracking after primary TKA with one of more malrotated components	Full-components revision (1 femoral component only)	37 (24–65)	All knees achieved normal patellar tracking	Mean Knee Society Score improved from 33 ± 18 preoperatively to 82 ± 6 at 6 months and 80 ± 8 at last follow-up. Only 1 patient complained of constant significant pain	1 undisplaced patella fracture treated conservatively; 1 pulmonary embolism
Pietsch et al. 2011 [59]	IV	14	64 (41–73)	Patella maltracking after primary TKA with isolated internal femoral component malrotation	Components revision (7 patients had also patella resurfacing)	57 (46–89)	All knees achieved normal patellar tracking	The mean Knee Society Score and Function increased from 52 (range 26–69) and 65 (range 30–90) to 85 (range 66–94) and 84 (range 65–100). The mean Hospital for Special Surgery Score increased from 63 (range 51–74) to 83 (range 68–91). 6 knees were rated excellent, 5 good, 1 fair	none
Van Gennip et al. 2012 [60]	IV	9	75 (60–83)	a. 6 Patella maltracking after primary TKA b. 3 Patella maltracking after revision TKA	- 7 MPFL reconstruction and lateral retinaculum release -2 MPFL reconstruction, lateral retinaculum release, and tibial tuberosity transfer	33 (10–48)	Median patellar displacement and tilt improved from 29 mm (range 0–44) and 45° (range 23–63) preoperatively to 0 mm (range 0–9) and 15° (range -3°-21°)	Median VAS satisfaction was 8 (range 5–9) and only one patient reported twice having a feeling related to subluxation	none
Goto et al. 2014 [61]	V	1	78	Patella maltracking after primary TKA	MPFL reconstruction and lateral retinaculum release	12	The knee achieved normal patellar tracking	The patient had clinical and functional improvement without a specific score system declared	none
Lamotte et al. 2016 [62]	IV	6	77 (70–87)	a. 4 Patella maltracking after primary TKA b. 2 Patella maltracking after primary TKA with components internally rotated	a. 4 MPFL reconstruction b. 1 MPFL reconstruction and components revision; 1 isolated MPFL reconstruction because of the high anesthesia risk	23(6–46)	Patellar tilt was less in all patients but 1, that had no change on the radiographs	None of the patients had a recurrence of the dislocation at the last follow-up and the functional scores (Kujala and subjective IKDC) improved in all patients except one, that had minimal clinical improvement and continued to experience pain with a feeling of patellar instability	none
Matar et al. 2020 [63]	IV	3	75,87,73	a. Patella maltracking after primary TKA with components internally rotated b, c. Patella maltracking after revision TKA	a. components revision and extensor mechanism reconstruction (elevate vastus lateralis off the intermuscular septum up to the mid-thigh, lateral retinaculum release, partial release of IT band, VMP advancement) b,c. extensor mechanism reconstruction (as a.) only	16,32,12	All knees achieved normal patellar tracking	Clinical and functional improvement with post-operative Knee Society Score of 92,85 and 79	none
Shen et al. 2020 [64]	V	1	84	Patella maltracking after primary TKA	Partial lateral patella facetectomy, lateral retinaculum release and vastus medialis restore	12	The knee achieved normal patellar tracking	Clinical and functional improvement with post-operative Hospital for Special Surgery Clinical Score of 85	none
Warschawski et al. 2020 [65]	IV	36	67 (49–78)	Patella maltracking after primary TKA with one of more malrotated components	Full-components revision	56 (5–145)	All knees achieved normal patellar tracking, except one patient, had a recurrent dislocation episode	Clinical and functional improvement with postoperative mean Knee Society Score of 86.2 at final follow-up	1 tibial tuberosity fracture
Saito et al. 2020 [66]	V	1	68	Patella maltracking after primary TKA with femoral component installed at a valgus position	Closing-wedge distal femoral varus osteotomy, MPFL reconstruction, lateral retinaculum release, patella resurfacing and substitution of the polyethylene insert	24	The knee achieved normal patellar tracking	The Kujala functional score and the Oxford knee score improved from 24 to 58 and from 28 to 40	none

^*^Patients were graded according to a knee rating system: 15 points were assigned for pain, 30 points for function, 10 points for deformity, and 10 points for motion. A score over 80 points represents a good result and over 90 points an excellent result

**Table 2:** Summary of the main surgical procedures used to treat the patella maltracking after TKA described in the included studies

Surgical procedure	Reference studies
Lateral retinaculum release	[9, 49, 50, , 51, 52, 18, 56, 57, 60, 61, 63]
Partial lateral patella facectomy	[64]
Medial relief (medial plication, advancement of VMO, V–Y quadricepsplasty)	[52, 18, 57, 63, 64]
MPFL reconstruction	[54, 60, 61, 52]
Extensor mechanism realignment: ◦ proximal realignment (Insall procedure) ◦ distal realignment: -ATT transposition (Hauser, Fulkerstone, Emslie-Trillat procedures) - Roux-Goldthwait procedures	[9, 48] [48, 51, 18, 53, 56, 57, 60] [53]
Prosthetic components revision	[9, 48, 18, 55, 56, 58, 59, 62, 63, 65]
Corrective osteotomy	[66, 52]

Most of the papers were rated as level IV according to the 2011 Oxford Center for Evidence-based Medicine Levels of Evidences; just two studies were rated as level V being case reports. All selected studies provide clinical, functional, and radiological results and complications concerning the management of patella maltracking after TKA, specifically in postoperative treatments.

## Discussion

Because nonsurgical treatments such as bracing and physical therapy after primary or revision TKA are not very effective to solve patella maltracking, surgical intervention is usually indicated [67].

First of all, it is crucial to identify the cause of the maltracking condition, to select the appropriate surgical procedure [60, 62, 65].

In the absence of components malpositioning, soft-tissue reconstructions of the extensor mechanism should be considered as a first measure to manage patella maltracking. Sometimes, a lateral retinaculum release could be enough to address patella maltracking [9, 18, 52], preserving the superior lateral geniculate vessels and performed step by step starting from the release of the lateral patellofemoral ligament and proceeding distally as long as needed [35, 39]. Bocell et al. [49], Johnson et al. [50] reported excellent results in patella tracking restoration performing an arthroscopic lateral release with the resolution of patellar symptoms. Nevertheless, performing an arthroscopy procedure after TKA is a technically challenging procedure and further studies are needed to define indications and expected results [49, 50]. A lateral retinaculum release is rarely performed alone and often is used together with other soft-tissue procedures as the advancement of the vastus medialis oblique or a balanced medial plication [18, 52, 63]. Furthermore, Chin et al. [52] reported a V–Y quadricepsplasty in severe cases of extensor mechanism tightness and Shen et al. [64] proposed a partial lateral patella facetectomy in addition to the lateral retinaculum release.

Whiteside et al. [53] suggest that simple lateral releases, medial plications and extra-articular vastus medialis advancements often are insufficient to correct mechanical patellar abnormalities and to prevent progressive subluxation; however, these techniques could be used as a first line of treatment or as a support to other surgical procedures.

During the 1990s, more attention was directed toward the medial patellofemoral ligament (MPFL) as one of the important medial stabilizers of the patella. Several biomechanical studies have demonstrated that the MPFL is the primary patella medial restraint, and a number of clinical studies have shown that patellar dislocation is often associated with injuries of the MPFL [60]. In TKA procedures, the medial structures can be damaged, due to the use of a medial parapatellar approach, patellar eversion and an inadequate closure, leading to patellar displacement [62]. Asada et al. [54], Goto et al. [61], Lamotte et al. [62], Van Gennip et al. [60] have demonstrated that the patellofemoral realignment procedure with MPFL reconstruction is an effective treatment for patellar symptomatic subluxation or dislocation after TKA in terms of achieving excellent results in the restore of clinical and radiographic patella tracking and improvement in functional scores, with minimum or null complications reported. MPFL reconstruction was carried out by authors using a quadriceps tendon split [60] or a tendon graft as semitendinosus [54, 60, 61], gracilis [62] or tibialis posterior [60]. Generally, the new MPFL is tightened from the medial side of the patella to a point between the adductor tubercle and the medial epicondyle: this technique guarantees the most anatomical and isometric reconstruction of the MPFL [60]. The reconstruction can be carried forward also through an extra-articular procedure as described by Asada et al. [54]: anchoring the graft to the distal site of the adductor tubercle and fixing the opposite end onto the proximal one-third of the patellar surface in an interlacing fashion, this technique allows to not open the joint capsule, with advantages in terms of reduction of surgical time and overall complications.

Patellofemoral realignment procedure with MPFL reconstruction is an effective treatment in patella maltracking exclusively in patients without malrotation of the prosthetic components. MPFL reconstruction should be performed only after a CT-scan, excluding abnormal internal rotation of the femoral component and/or pathological TT-TG distance, that could make the MPFL reconstruction alone ineffective for the restore of correct patellar tracking [54, 60–62].

Extensor mechanism realignment procedures (proximal, distal or combined) have been proposed by several authors in the last decades. Merkow et al. [9] and Grace et al. [48] have managed patients with patella maltracking after TKA performing a proximal realignment procedure as illustrated by Insall, achieving optimal results. This technique consist in an exposure of the quadriceps mechanism through a midline skin incision and two deep capsular incisions, one medial and the other lateral. Realignment is accomplished by advancing the medial flap containing the vastus medialis, laterally and distally in line with the fibers of the oblique portion of the vastus medialis over the anterior surface of the patella. After suturing the edge of the advanced medial flap in place near the lateral margin of the patella, the suture is completed along the front of the patella and the lateral release is performed. This realignment technique corrects patellar tilt and decreases the functional quadriceps angle, changing the direction of pull of the quadriceps muscle [9]. Nevertheless, in case of increased Q-angle as main etiologic cause of the patella maltracking, distal realignment procedures are preferable, even as additional procedure if the proximal realignment appears insufficient to restore good patellar tracking [51, 53].

Several distal realignment techniques were described to manage the patella maltracking. Procedures as the Hauser [48], Emslie-Trillat [18, 51, 53, 57] and Fulkerson [56] consist in a medial and variable distal displacement of the tibial tubercle, secured with screws or wires to the tibial cortex, while Roux-Goldthwait techniques [53] consist in a medial transferring of the lateral or medial ½ of the patellar tendon, sutured directly to the capsular edge. Notable works developed by Grace et al.[48], Kirk et al. [51], Campbell et al. [18], Whiteside et al. [53], Price et al. [56], Nakajima et al. [57], Van Gennip et al. [60] proposed these distal realignment procedures to treat patella maltracking after TKA with optimal results. Distal realignment procedures featuring a tibial tubercle transfer should be selected with caution in patients with osteoporotic tibial metaphysis or poor tibial bone stock as in revision TKAs. In fact, in these cases, there is a greater risk of complications as nonunion or osteonecrosis of the tibial tubercle fragment with loss of staple fixation, patellar tendon rupture, variable loss of flexion arch, and hematomas that could lead to a wound or deep infection [9, 48, 51, 53]. Furthermore, the fixation of the tubercle fragment in revision TKAs could be hard considering the stemmed prosthetic implant [51, 56]. Distal realignment techniques that do not use tibial tubercle transfer as the Roux-Goldthwait appear to have lower complication rates: Whiteside et al. [53] reported no significant patellar complications occurred in patients who underwent these procedures, even if may appear insufficient to restore an adequate patella tracking in case of severe patellar subluxation or persistent dislocation.

Another limitation of tibial tubercle transfer techniques is an inability to perfectly normalize the patellar tilt, which, however, not significantly affect the clinical outcome [57].

Patella maltracking after TKA is often due to malrotated prosthetic components. Incavo et al. [55], Lakstein et al. [58], Pietsch et al. [59], Warschawski et al. [65] and other authors largely demonstrated that patients with painful TKAs resulting from components malrotation can achieve symptomatic improvement with revision surgery, preferably within 2 years from the primary procedure. Authors suggest also replacing the patella even if it has not been performed in the primary arthroplasty procedure, especially if its thickness is adequate and better following procedural helpful measures such as the medialization of the patellar button [18, 55, 59].

Only a CT scan should be considered an accurate method to diagnose and quantify the degree of rotational malalignment of the prosthetic components [58, 59, 65]. Despite the consensus between authors to consider the tibial component as internal rotated if its antero-posterior axis is directed medial to the medial third of the tibial tubercle [55], agreement regarding the definition of the femoral prosthetic component as internal rotated remains unclear. The cut-off angle was defined in relation to the posterior condylar angle as 4° according to Pietsch et al. [59] and 3° to Lakstein et al. [58], while Incavo et al. [55] proposed a femoral component as internal rotated above 5° from the epicondylar axis.

Authors suggest that complete revision leads to better clinical results compared to a partial revision, even in cases of isolated femoral internal malrotation [55, 59, 65]. In fact, if the femoral component is internally rotated, there are generally consequences regarding altered flexion spaces with a tight internal compartment and a mediolateral soft-tissue imbalance, difficult to restore only with a singular component revision. Also, the relatively good results after revision surgery in these procedures often require the use of a constrained condylar prostheses: this cannot be used if the original tibial component is left in place [55].

Considering the malpositioning of prosthetic components, Saito et al. [66] described a successful treatment of an habitual patellar dislocation after a TKA with the femoral component implanted at an extremely valgus position using a medial closing-wedge distal femoral osteotomy, reporting optimal patella tracking correction and patient’s clinical satisfaction. Nevertheless, further studies are needed to define osteotomy procedures as efficient and reproducible techniques to manage patella maltracking in case of axial malpositioning of the prosthetic components.

A meta-analysis could not be performed from this systematic review because of the insufficient statistical power correlated to the low number of cases in some of the included studies and the heterogeneity related to the different clinical and functional score system used by authors to describe their results. Furthermore, five of the included studies have not declared any specific score system and feature only a qualitative descriptions of the results. It could be also difficult to fully compare the results of patella maltracking correction without a cases homogenization about knee prosthetic model and alignment method adopted.

A last limitation of this review concerns the use in many included works of combined procedures to manage the patella maltracking; thus, it could be difficult in certain articles to interpret the relative contribution of single techniques in the final correction result obtained.

## Conclusion

This review of the literature describes in a systematic collection the main techniques used by authors to approach patella maltracking after total knee arthroplasty, representing a frequent and hard to manage problem for orthopedic surgeons. Treatments described by authors range from simple soft-tissue procedures to complete prosthetic components revision. The indication of a specific technique depends on several factors, first of all the eventual malpositioning of prosthetic components. Furthermore, paying attention to the patient’s clinical characteristics as osteoporosis, vascular suffering conditions or having an already revisioned knee could be helpful in the choice of proper procedures.

Although these established operative techniques are able to correct an abnormal patellar tracking, as proved by radiograms and clinical evidences of right patellofemoral relationship in the flexo-extension arch, authors report constantly variable percentages of residual knee pain and dissatisfaction in re-treated patients.

Since it is largely demonstrated that patella maltracking is generally a consequence of intra-operative technical errors, it is desirable to prevent maltracking conditions at the time of primary arthroplasty using proper surgical precautions, especially for the emotional and financial costs derived from a revision procedure.

## Data Availability

Not applicable.
